# 1,25(OH)_2_D_3_ Mitigates Oxidative Stress-Induced Damage to Nucleus Pulposus-Derived Mesenchymal Stem Cells through PI3K/Akt Pathway

**DOI:** 10.1155/2022/1427110

**Published:** 2022-03-18

**Authors:** Jun-wu Wang, Lei Zhu, Peng-zhi Shi, Ping-chuan Wang, Yan Dai, Yong-xiang Wang, Xu-hua Lu, Xiao-fei Cheng, Xin-min Feng, Liang Zhang

**Affiliations:** ^1^Department of Orthopedics, Clinical Medical College of Yangzhou University, Yangzhou 225001, China; ^2^Graduate School of Dalian Medical University, Dalian 116000, China; ^3^Medical Experimental Research Center, Clinical Medical College of Yangzhou University, Yangzhou 225001, China; ^4^Department of Orthopedics, Changzheng Hospital of The Second Military Medical University, Shanghai 200003, China; ^5^Department of Orthopedic Surgery, Shanghai Key Laboratory of Orthopedics Implants, Shanghai Ninth People's Hospital, Shanghai Jiaotong University School of Medicine, Shanghai 200011, China

## Abstract

Intervertebral disc degeneration (IVDD) is one of the main causes of low back pain. The local environment of the degenerated intervertebral disc (IVD) increases oxidative stress and apoptosis of endogenous nucleus pulposus-derived mesenchymal stem cells (NPMSCs) and weakens its ability of endogenous repair ability in degenerated IVDs. A suitable concentration of 1*α*,25-dihydroxyvitamin D_3_ (1,25(OH)_2_D_3_) has been certified to reduce oxidative stress and cell apoptosis. The current study investigated the protective effect and potential mechanism of 1,25(OH)_2_D_3_ against oxidative stress-induced damage to NPMSCs. The present results showed that 1,25(OH)_2_D_3_ showed a significant protective effect on NPMSCs at a concentration of 10^−10^ M for 24 h. Protective effects of 1,25(OH)_2_D_3_ were also exhibited against H_2_O_2_-induced NPMSC senescence, mitochondrial dysfunction, and reduced mitochondrial membrane potential. The Annexin V/PI apoptosis detection assay, TUNEL assay, immunofluorescence, western blot, and real-time quantitative polymerase chain reaction assay showed that pretreatment with 1,25(OH)_2_D_3_ could alleviate H_2_O_2_-induced NPMSC apoptosis, including the apoptosis rate and the expression of proapoptotic-related (Caspase-3 and Bax) and antiapoptotic-related (Bcl-2) proteins. The intracellular expression of p-Akt increased after pretreatment with 1,25(OH)_2_D_3_. However, these protective effects of 1,25(OH)_2_D_3_ were significantly decreased after the PI3K/Akt pathway was inhibited by the LY294002 treatment. *In vivo*, X-ray, MRI, and histological analyses showed that 1,25(OH)_2_D_3_ treatment relieved the degree of IVDD in Sprague–Dawley rat disc puncture models. In summary, 1,25(OH)_2_D_3_ efficiently attenuated oxidative stress-induced NPMSC apoptosis and mitochondrial dysfunction via PI3K/Akt pathway and is a promising candidate treatment for the repair of IVDD.

## 1. Introduction

The prevalence of low back pain (LBP) is 11.9%, as reported in the *Lancet*, and the peak prevalence ranges from 28% to 42% in people of middle- to old-age (40~69 years) [1]. Intervertebral disc degeneration (IVDD) is a usual cause of LBP in aging people and increases the socioeconomic burden [2, 3]. Regrettably, conventional treatments mainly relieve symptoms instead of repairing or regenerating the structure and function of the degenerative intervertebral disc (IVD) [4]. In recent years, multiple types of mesenchymal stem cells (MSCs), including bone marrow-derived MSCs (BMSCs), adipose-derived MSCs, umbilical cord-derived MSCs, and other types of MSCs have been used to treat IVDD [5–9].The separation and identification of endogenous nucleus pulposus-derived MSCs (NPMSCs) in 2007 rendered a possibility for the endogenous restoring of IVDD [10]. Our previous study also confirmed that NPMSCs exist in normal and degenerated IVDs [11]. Interestingly, compared with MSCs from other sources, NPMSCs have better tolerance in the hyperosmolar and acidic microenvironment of deteriorated IVDs [12]. However, pathological factors, including annulus fibrosus (AF) rupture, inflammation, and oxidative stress, may induce the generation of reactive oxygen species (ROS) [13]. The generated ROS enhances the senescence and apoptosis of nucleus pulposus (NP) cells and NPMSCs, which are primary characteristics of IVDD [14, 15].

The proliferation and migratory ability of BMSCs were enhanced after treatment with 1*α*,25-dihydroxyvitamin D_3_ (1,25(OH)_2_D_3_) via activation of thePI3K/Akt pathway [16]. Vitamin D was found can participate in the proliferation, aging, apoptosis, inflammation, oxidative stress, and extracellular matrix expression of NP cells [17]. Moreover, previous studies including our study found that the phosphatidylinositide 3-kinases/protein kinase B (PI3K/Akt) pathway is closely related to NPMSC apoptosis [18–20]. It has also been reported that 1,25(OH)_2_D_3_ inhibits cell apoptosis through the PI3K/Akt pathway [21, 22]. Therefore, the purpose of this study was to explore whether 1,25(OH)_2_D_3_ protects NPMSCs from H_2_O_2_-induced oxidative damage via PI3K/Akt pathway and to provide a basis for its clinical application in the treatment of IVDD.

## 2. Materials and Methods

### 2.1. Isolation and Culture of NPMSCs

Sprague–Dawley (SD) rats (weight, 250-350 g; age, 2.5–3.5 months) were purchased from the Laboratory Animal Center of Yangzhou University (License No. SYXK (Su) 2021–0027). The Ethical Committee of the Clinical Medical College of Yangzhou University approved the experimental studies. The separation method of NP tissues was the same as described in our previous studies [11, 18, 23–26]. Briefly, the SD rats were euthanized with an anesthesia overdose, and the collected NP tissues from the SD rats were separated under aseptic conditions and washed carefully with phosphate-buffered saline (PBS) containing 1% penicillin–streptomycin (Gibco, USA). The NP tissues were cut into fragments of 1 mm^3^ and digested by collagenase type II (Gibco, USA) for 12 h at 37°C. After being washed with normal saline and centrifuged at 1000 rpm for 4 min, the cells were resuspended in MSC complete medium (Cyagen, USA) complemented with 10% fetal bovine serum (FBS; HyClone, USA) and 1% penicillin–streptomycin and cultured at 37°C under 5% CO_2_. The culture medium was replaced every three days. Each primary culture was digested and subcultured at a ratio of 1 : 3 when the adherent cells met 80% confluence. A microscope (Olympus, Japan) was used to observe and photograph the cells. Third passage cells were gained for subsequent experiments.

### 2.2. Surface Marker Identification of NPMSCs

Surface markers, including CD34, CD45, CD73, CD90, and CD105 have been reported to identify MSCs according to the standards proposed by the International Society for Cellular Therapy (ISCT) [27]. Immunofluorescent staining was used to examine these surface markers. Briefly, NPMSCs were inoculated on 25 mm diameter polylysine-containing cell slides in a 12-well plate and cultured in the MSC complete medium. NPMSCs were then fixed with 4% paraformaldehyde for 15 min and washed twice with PBS containing 0.5% Triton X-100 for 15 min. After being blocked with 10% bovine serum albumin for 1 h at 37°C, the cells were incubated with primary antibodies (1 : 100) at 4°C overnight. The cell slides were washed twice with TBST and then incubated with a conjugated secondary antibody (1 : 500; Abcam, United Kingdom, catalog Nos. ab150077, ab150078, ab150080, and ab175471) for 2 h at room temperature. After being treated with the antifade mounting medium containing 4′,6-diamidino-2′-phenylindole (DAPI) for 10 min, the cell slides were observed and photographed using a fluorescence microscope (Leica, Wetzlar, Germany).

### 2.3. Multilineage Differentiation Ability of NPMSCs

Osteogenic, adipogenic, and chondrogenic differentiation of NPMSCs was induced to identify its multilineage differentiation potential. Briefly, NPMSCs were seeded in 6-well plates, and multilineage differentiation kits (Cyagen Biosciences, China) were used following the manufacturer's instructions to induce cell differentiation when the cells reached 80% confluence. Subsequently, the cells were washed with PBS and fixed with 4% paraformaldehyde for 30 min when the required days for induction were reached. The cells were then stained with Alizarin red (Sigma, USA), Oil Red O (Sigma, USA), and Alcian blue (Sigma, USA) and recorded under a microscope.

### 2.4. Application of H_2_O_2_ and 1,25(OH)_2_D_3_ to NPMSCs

To explore the protective effect of 1,25(OH)_2_D_3_ on H_2_O_2_-induced damage, a dose- and time-response study with various concentrations of H_2_O_2_ (Jiancheng, China) (0~200 *μ*M, 0~6 h) and 1,25(OH)_2_D_3_ (MedChemExpress, USA) (0, 10^−12^, 10^−11^, 10^−10^, 10^−9^, 10^−8^, 10^−7^, 10^−6^, and 10^−5^ M, 0~48 h) was performed to determine the suitable protective dose and time of 1,25(OH)_2_D_3_. Based on the results (detailed in “Results”), NPMSCs were preconditioned with 10^−10^ M 1,25(OH)_2_D_3_ for 24 h before exposure to 100 *μ*M H_2_O_2_ for 6 h in subsequent experiments. NPMSCs were preconditioned with LY294002 (a selective inhibitor of PI3K, MedChemExpress, USA) before H_2_O_2_ for 2 h at a concentration of 25 *μ*M to further test the association between the protective effects of 1,25(OH)_2_D_3_ and the PI3K/Akt pathway. The NPMSCs were divided into the following groups: (A) control group (blank), (B) H_2_O_2_ group (100 *μ*M H_2_O_2_), (C) 1,25(OH)_2_D_3_ group (10^−10^ M 1,25(OH)_2_D_3_+100 *μ*M H_2_O_2_), and (D) LY group (10^−10^ M 1,25(OH)_2_D_3_+25 *μ*M LY294002+100 *μ*M H_2_O_2_).

### 2.5. Cell Viability Assay

A cell counting kit-8 (CCK-8, Beyotime, China) was used to detect the cytotoxicity of 1,25(OH)_2_D_3_ on NPMSCs. Briefly, NPMSCs were seeded into 96-well plates at a density of 5 × 10^3^ cells/well and cultured at 37°C under 5% CO_2_. Then, 10 *μ*l CCK-8 solution was added to each well after being separately treated as described above for 24 h. The optical density (OD) value was measured at 450 nm with a microplate reader (Bio–Rad, Hercules, United States) after 2 h. The cell viability was calculated as follows: cell viability (100% of control) = [(Ae − Ab)/(Ac − Ab)] × 100%, where Ae, Ab, and Ac represent the A450 of the treatment, blank, and control groups, respectively.

### 2.6. 5-Ethynyl-2′-Deoxyuridine (EdU) Incorporation Assay

An EdU cell proliferation detection kit (Beyotime, China) was used to detect cell proliferation. The NPMSCs were seeded into 6-well plates at a density of 4 × 10^4^ ~ 5 × 10^4^ cells/well at 37°C under 5% CO_2_. After incubated with EdU for 2 h, the NPMSCs were fixed with 4% paraformaldehyde for 15 min, permeated with 0.5% Triton X-100 for 10 min, and incubated with Click Reaction Mixture for 30 min, successively. The cells were then washed with PBS and counterstained with Hoechst 33342 in the dark. A fluorescence microscope and ImageJ software (NIH, USA) were used to determine and analyze the number of EdU-positive nuclei.

### 2.7. Senescence *β*-Galactosidase (SA-*β*-Gal) Staining

An SA-*β*-Gal staining kit (Beyotime, China) was used to detect SA-*β*-gal activity in NPMSCs according to the manufacturer's instructions. The NPMSCs were inoculated into 6-well plates at the density of 4 × 10^4^ ~ 5 × 10^4^ cells/well at 37°C under 5% CO_2_. Briefly, after being washed with PBS, the cells were fixed in an SA-*β*-gal fixative solution for 15 min at room temperature. Rewashed 3 times with PBS, the cells were then incubated with an SA-*β*-gal working solution overnight at 37°C without CO_2_. Afterward, the NPMSCs were observed under a microscope and analyzed using the ImageJ software.

### 2.8. Annexin V-FITC/PI Staining

An Annexin V-FITC/PI apoptosis detection kit (KeyGen, China) was used to analyze cell apoptosis. After being washed with PBS, the NPMSCs of each group were collected by trypsinization and resuspended in 500 *μ*l of 1x binding buffer. Then, 5 *μ*l PI and 5 *μ*l Annexin-V were added in cell suspension, and the cells were incubated in the dark at room temperature for 15 min. The percentage of cells positively labeled by Annexin V-FITC and PI was analyzed using flow cytometry (BD Company, USA).

### 2.9. TUNEL Staining

The level of DNA damage was evaluated using terminal deoxynucleotidyl transferase-mediated dUTP nick end labeling (TUNEL) staining (Beyotime, China). The NPMSCs from the four groups were fixed in freshly prepared 4% paraformaldehyde for 30 min at 37°C, permeabilized with 0.1% Triton X-100 for 5 min, and washed with PBS 3 times at each step. Afterward, the cells were stained with TUNEL according to the manufacturer's instructions, and a fluorescence microscope and ImageJ software were used to observe and analyze the number of TUNEL-positive cells.

### 2.10. JC-1 Assay for Mitochondrial Membrane Potential (MMP)

The MMP of NPMSCs was detected using a fluorescent probe iodide 5,5′,6,6′-tetrachloro-1,1′,3,3′-tetraethylbenzimidazolcarbocyanine (JC-1) detection kit (KeyGen, China). In normal mitochondria, JC-1 aggregates in the mitochondrial matrix to form a polymer, which emits intense red fluorescence (polarized). In damaged mitochondria, JC-1 exists in the cytoplasm in the form of a monomer and produces green fluorescence (depolarization) due to the decrease or loss of MMP. After treatment according to the manufacturer's instructions, the NPMSCs in each group were observed and photographed using a fluorescence microscope. The ratio of green to red fluorescence intensity was analyzed using the ImageJ software.

### 2.11. ROS Assay

A ROS detection fluorescent probe dihydroethidium (DHE) kit (KeyGen, China) was used to measure the generation of ROS in NPMSCs. The NPMSCs were inoculated into a 6-well plate and treated as described above. The NPMSCs were then washed twice with PBS and incubated with 10 *μ*M DHE for 30 min at 37°C. A fluorescence microscope and ImageJ software were used to observe and analyze the intracellular ROS fluorescence.

### 2.12. Immunofluorescence Assay

The intracellular expression of the target proteins was detected using immunofluorescence staining. Briefly, NPMSCs were inoculated on 25 mm diameter polylysine-containing cell slides in a 12-well plate and treated as described above. The NPMSCs were then fixed in 4% paraformaldehyde for 15 min and washed twice with PBS containing 0.5% Triton X-100 for 15 min at room temperature. The NPMSCs were then blocked with QuickBlock™ Blocking Buffer for Immunol staining (Beyotime, China) for 1 h. The cells were incubated with primary antibodies (including p-Akt (bs-0876R), Bioss, China; Akt (#4691), Cell Signaling Technologies, USA; p21 (10355-1-AP) and p53 (10442-1-AP), Proteintech, USA; and Caspase-3 (A0214), Bax (A0207), and Bcl-2 (A11025), ABclonal, USA; 1 : 100) overnight at 4°C in a dark wet chamber. Afterward, the cells were washed twice with TBST and incubated with a conjugated goat anti-rabbit IgG secondary antibody at 37°C for 2 h. Subsequently, after being stained with DAPI for 10 min, the stained NPMSC samples were visualized and photographed using a fluorescence microscope or a laser scanning confocal microscope (Zeiss LSM 710, Germany) and analyzed using the ImageJ software.

### 2.13. Western Blot Assay

A Whole Cell Lysis Assay Kit (KeyGen, China) was used to extract the total proteins, and a BCA protein assay kit (Beyotime, China) was then used to measure the protein concentration. Equal protein samples from each group were separated by 10% SDS–PAGE gels and then transferred to PVDF membranes (Millipore, USA). Subsequently, the membranes were blocked in 5% nonfat milk 2 h on a shaker at room temperature and incubated with primary antibodies overnight at 4°C. The membranes were then washed with TBST three times and incubated with secondary antibodies for 2 h at room temperature. Afterward, protein signals were visualized using an enhanced chemiluminescence system and analyzed using the ImageJ software. The loading control was *β*-actin.

### 2.14. Real-Time Quantitative Polymerase Chain Reaction (qRT–PCR) Assay

The mRNA expression levels of Caspase-3, Bcl-2, Bax, and *β*-actin were quantified. The total RNA of the NPMSCs was extracted using TRIzol reagent (Invitrogen, USA). According to the manufacturer's instructions, reverse transcription from the whole RNA to complementary DNA (cDNA) was performed using Prime Script-RT reagent kit (Vazyme Biotech, China), and amplification of cDNA was performed using SYBR Premix Ex Taq (Vazyme Biotech, China). The comparative Ct method was used to calculate the expression levels of target genes in the different groups. *β*-Actin expression levels were used to normalize relative gene expression levels. The primers were designed according to the sequences in GenBank using the Prime 5.0 software and are listed in Table 1.

### 2.15. IVDD Model Induction

Eighteen SD rats were randomly divided into three groups (*n* = 6 per group): control group (no operation), IVDD group (punctured), and 1,25(OH)_2_D_3_ group (punctured and treated with 1,25(OH)_2_D_3_). The IVDD model was established as reported previously [28]. Briefly, the rat was placed in a prone position after anesthesia with an intraperitoneal injection of 1% pentobarbital sodium at 0.1 mg/kg, and a percutaneous needle puncture was performed with a 21G needle in the coccygeal intervertebral disc (Co 6-7). The needle was carefully inserted in the middle of the disc, perpendicular to the skin and parallel to the endplate, rotated 180°, and held for 5 s. After removal of the needle, the wound was covered with gauze and the rats underwent standard postoperative procedures. The 1,25(OH)_2_D_3_ was dissolved in DMSO (10^−3^ M/L) and further diluted in saline immediately before intraperitoneal administration. Two weeks of puncture, the 1,25(OH)_2_D_3_ group received 100 ng/kg/day 1,25(OH)_2_D_3_ [29]. Meanwhile, the control group and IVDD group received an equal amount of saline supplemented with the required volume of DMSO.

### 2.16. X-Ray and Magnetic Resonance Imaging (MRI) Assay

X-ray and MRI scans were taken prepuncture and at 6 weeks after the puncture, respectively. X-ray was used to measure the disc height index (DHI) [30]. A 7.0-T MRI scanning system (Philips Intera Achieva 7.0 MR, Netherlands) was used, and sagittal T2-weighted images were used to assess the Pfirrmann grade of IVDD based on signal intensity and intervertebral disc height [31].

### 2.17. Histologic Assay

All rats were euthanized by an anesthesia overdose after 4 weeks of treatment. The harvested IVD specimens were fixed with 4% paraformaldehyde, decalcified with a 10% ethylenediaminetetraacetic acid solution, and embedded in paraffin, successively. The specimens were then cut into 5 *μ*m slices, and the slices were stained with hematoxylin-eosin (HE), toluidine blue, and safranin-O/fast green (S-O). The histologic grading scale criteria reported by Norcross et al. [32] was adopted to evaluate the histologic images.

### 2.18. Statistical Analysis

Analyses were performed using IBM SPSS 19.0, and GraphPad Prism 8 was used to make statistical graphs. All measurements were performed in triplicate, and the results are expressed as the mean ± standard deviation (mean ± SD). The Kolmogorov–Smirnova test or Shapiro–Wilk test is used to check whether the data conformed to a normal distribution. One-way analysis of variance (ANOVA) was used to analyze the data from multiple groups. The Student *t*-test was applied to analyze the differences between the two groups. *P* < 0.05 was considered statistically significant.

## 3. Results

### 3.1. Identification of NPMSCs

**Figure 1:**
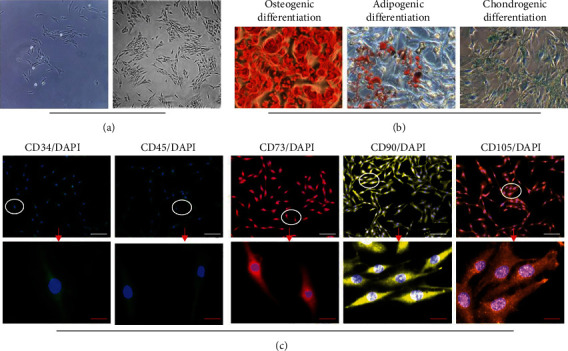
Identification of nucleus pulposus-derived mesenchymal stem cells (NPMSCs). (a) NPMSCs present with a long spindle shape and grow in a flower formation (scale bar = 100 *μ*m). (b) NPMSCs exhibit a low fluorescence expression of the hematopoietic stem cell surface markers CD34 and CD45, but a high fluorescence expression of the MSC surface markers CD73, CD90, and CD105; (c) NPMSCs are positive for Alizarin red, Oil Red O, and Alcian blue staining after multilineage differentiation (white scale bar = 50 *μ*m; red scale bar = 20 *μ*m).

The separated NPMSCs gathered into a chrysanthemum shape and formed into a long spindle shape after passage (Figure 1(a)). Similar to our previous studies [18, 23, 33], the MSC-associated surface markers CD73, CD90, and CD105 in NPMSCs presented high fluorescence expression, whereas the surface markers CD34 and CD45 showed presented low fluorescence expression (Figure 1(b)). The results of osteogenic, adipogenic, and chondrogenic induction *in vitro* confirmed the multipotential differentiation of NPMSCs (Figure 1(c)). These results demonstrated that the NPMSCs isolated from the IVD corresponded to the criteria of MSC proposed by ISCT.

### 3.2. Ideal Incubation Concentrations of H_2_O_2_ and 1,25(OH)_2_D_3_

**Figure 2:**
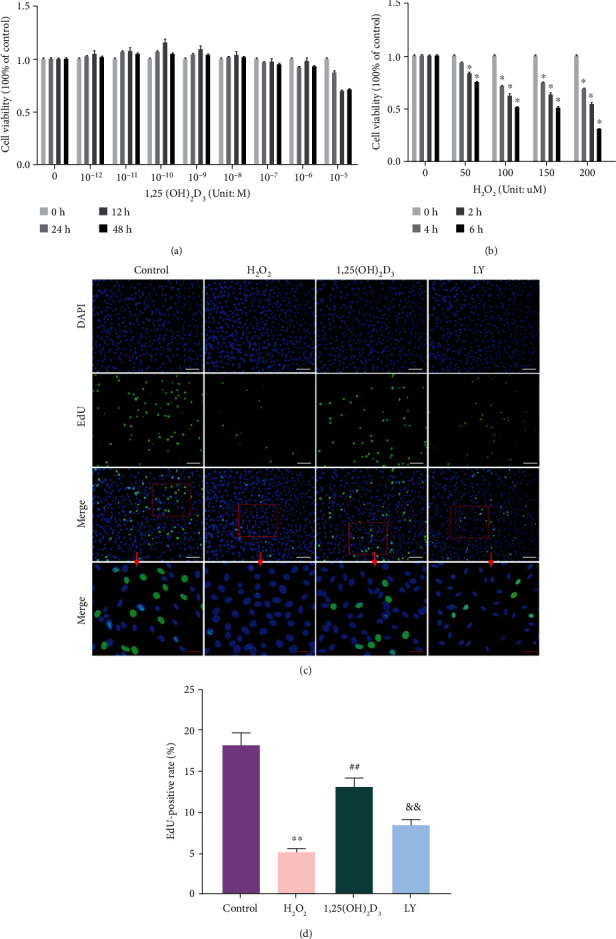
(a) 1,25(OH)_2_D_3_ shows no cytotoxicity effect on cell viability at a concentration of 10^−8^ M to 10^−12^ M for 24 h. (b) 100 *μ*M H_2_O_2_ treatment for 6 h leads to appropriate inhibition of cell proliferation. (c) EdU assay results of nucleus pulposus-derived mesenchymal stem cells (NPMSCs) in the different groups. Green fluorescence represents cells in a proliferating state (white scale bar = 100 *μ*m; red scale bar = 25 *μ*m). (d) Quantitative analysis of EdU-staining positive cells. All data are the means ± SD of at least three independent experiments. ^∗^*P* < 0.05 and ^∗∗^*P* < 0.01 compared with the control group; ^##^*P* < 0.01 compared with the H_2_O_2_ group; ^&&^*P* < 0.01 compared with the 1,25(OH)_2_D_3_ group. LY: LY294002 group.

The viability effects of 1,25(OH)_2_D_3_ on NPMSCs were analyzed using a CCK-8 assay. The results showed that 1,25(OH)_2_D_3_ showed no cytotoxic effect on cell viability at a concentration of 10^−8^ M to 10^−12^ M for the different exposure times (0, 12, 24, and 48 h) (Figure 2(a)); 10^−10^ M of 1,25(OH)_2_D_3_ was chosen for the subsequent experiments. Additionally, cell proliferation was appropriately inhibited when treated with 100 *μ*M H_2_O_2_ for 6 h, which was the same result as our previous study [24, 33] (Figure 2(b)). Therefore, this condition was used to induce oxidative stress damage in NPMSCs in the following experiments.

### 3.3. 1,25(OH)_2_D_3_ Enhanced Cell Proliferation

The effect of 1,25(OH)_2_D_3_ on the proliferation of NPMSCs was detected using an EdU staining kit. As shown in Figures 2(c) and 2(d), the number of EdU-labeled NPMSCs in the H_2_O_2_ group was significantly lower than that in the control group (*P* < 0.05), and the number of EdU-labeled NPMSCs was partially increased in the 1,25(OH)_2_D_3_ group (*P* < 0.05). However, LY294002 significantly weakened the increased proliferation effect of 1,25(OH)_2_D_3_ (*P* < 0.05).

### 3.4. 1,25(OH)_2_D_3_ Inhibited Cell Senescence

**Figure 3:**
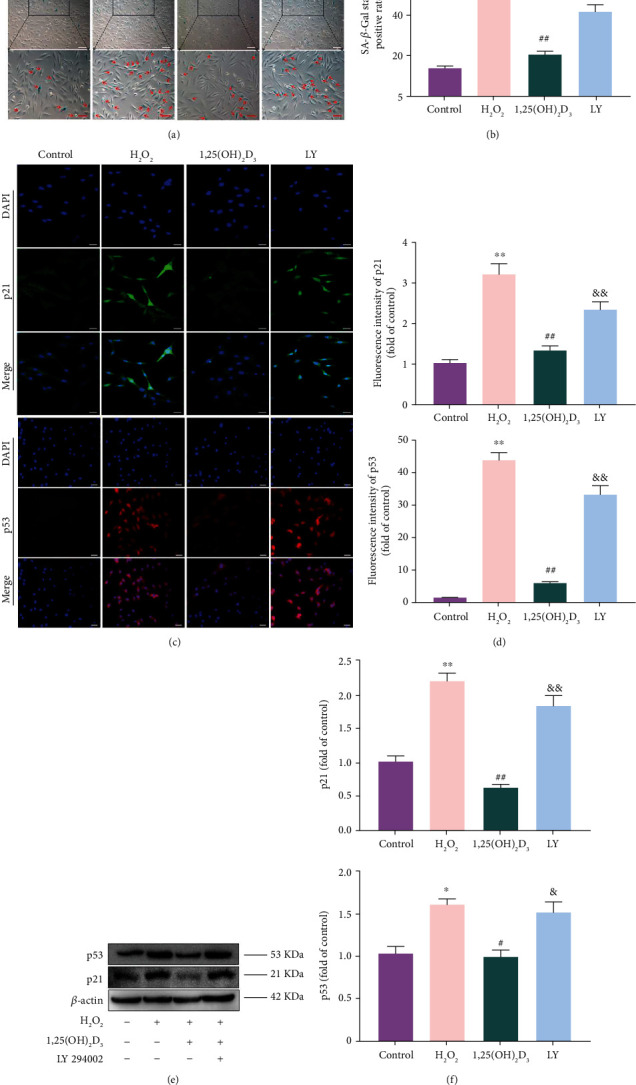
SA-*β*-gal staining assay and the expression levels of p21 and p53. (a) SA-*β*-gal staining results of nucleus pulposus-derived mesenchymal stem cells (NPMSCs) in the different groups. Aging cells displayed high expression of SA-*β*-gal in blue staining (white scale bar = 100 *μ*m; red scale bar = 50 *μ*m). (b) Quantitative analysis of SA-*β*-staining results. (c) Immunofluorescence staining of p21 is observed by a laser scanning confocal microscope, and the immunofluorescence staining of p53 is observed by a fluorescence microscope (scale bar = 25 *μ*m). (d) Quantitative analysis of the fluorescence intensity of p21 and p53. (e, f) The protein expressions and quantitative analysis of p21 and p53. All data are expressed as the mean ± SD. ^∗∗^*P* < 0.01 compared with the control group; ^##^*P* < 0.01 compared with the H_2_O_2_ group; ^&&^*P* < 0.01 compared with the 1,25(OH)_2_D_3_ group. LY: LY294002 group.

The cell senescence was evaluated using SA-*β*-Gal staining. Aging cells presented high SA-*β*-gal activity and stained blue. The percentage of SA-*β*-gal-positive NPMSCs in the H_2_O_2_ group was higher than that in the control group (*P* < 0.05). However, the percentage of SA-*β*-gal-positive NPMSCs was inhibited after 1,25(OH)_2_D_3_ pretreatment (*P* < 0.05). Incubation with LY294002 significantly weakened the protective effect of 1,25(OH)_2_D_3_, and the percentage of SA-*β*-Gal-positive cells was increased (*P* < 0.05, Figures 3(a) and 3(b)).

To further explore the active therapeutic effect of vitamin D on cell aging, we detected the expression of senescence-related proteins (p21 and p53) using immunofluorescence and western blotting. As shown in Figures 3(c)–3(f), the intracellular fluorescence and expression levels of p21 and p53 in NPMSCs of the H_2_O_2_ group were higher than those in the control group (*P* < 0.05), whereas the high fluorescence and increased protein expression levels of p21 and p53 were significantly reversed by pretreatment with 1,25(OH)_2_D_3_ (*P* < 0.05). However, pretreatment with LY294002 decreased the protective effect of 1,25(OH)_2_D_3_ by upregulating the expression levels of p21 and p53 in NPMSCs (*P* < 0.05). These results demonstrate that 1,25(OH)_2_D_3_ can inhibit the senescence of NPMSCs that is induced by H_2_O_2_.

### 3.5. 1,25(OH)_2_D_3_ Decreased H_2_O_2_-Induced Cell Apoptosis

**Figure 4:**
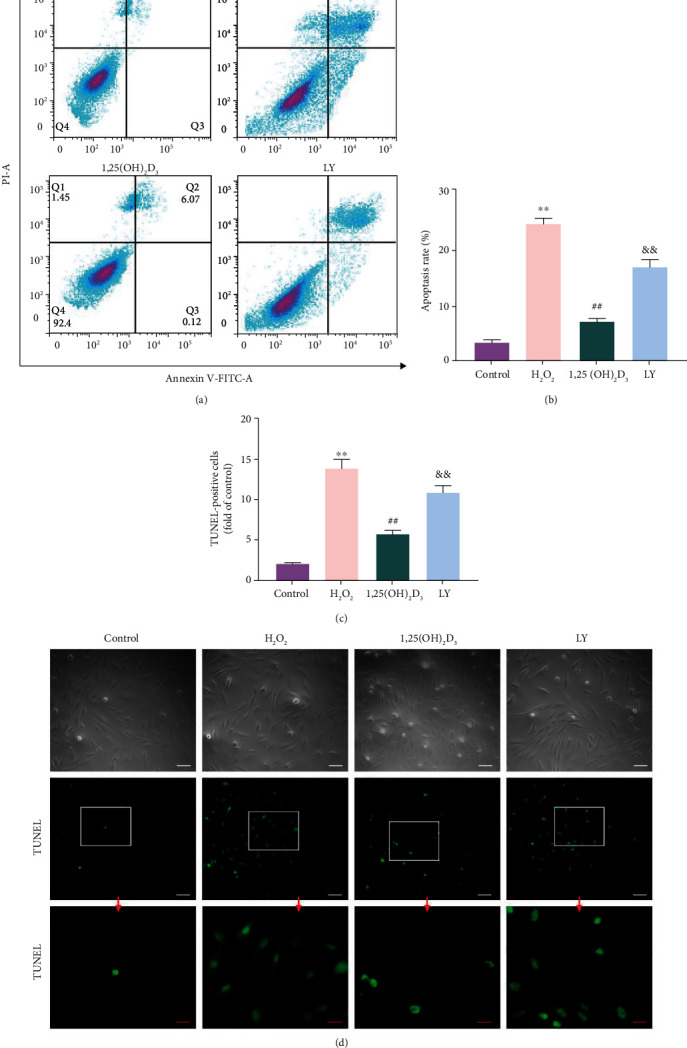
1,25(OH)_2_D_3_ improves H_2_O_2_-induced nucleus pulposus-derived mesenchymal stem cell (NPMSC) apoptosis. (a) Annexin V/PI staining flow cytometry results of NPMSCs. (b) Quantitative analysis of the apoptotic rate of NPMSCs. (c) TUNEL assay results of NPMSCs (white scale bar = 50 *μ*m; red scale bar = 25 *μ*m). (d) Quantitative analysis of TUNEL-staining positive cells. All data are the mean ± SD. ^∗∗^*P* < 0.01 compared with the control group; ^##^*P* < 0.01 compared with the H_2_O_2_ group; ^&&^*P* < 0.01 compared with the 1,25(OH)_2_D_3_ group. LY: LY294002 group.

The protective effect of 1,25(OH)_2_D_3_ against H_2_O_2_-induced apoptosis was evaluated using Annexin V/PI staining. Flow cytometry showed that more cells appeared in the Q2 and Q3 quadrants in the H_2_O_2_ group, which indicates that the apoptotic rate of NPMSCs in the H_2_O_2_ group was significantly higher than that in the control group (*P* < 0.05). The apoptotic rate of NPMSCs was partially decreased after pretreatment with 1,25(OH)_2_D_3_ (*P* < 0.05), but the presence of LY294002 significantly weakened the protective effect of 1,25(OH)_2_D_3_ (*P* < 0.05, Figures 4(a) and 4(b)).

The TUNEL assay was also used to evaluate cell apoptosis. The DNA in the apoptotic cell nucleus was broken, and the exposed 3′-OH linked to fluorescein-dUTP under the catalysis of terminal deoxynucleotidyl transferase, which causes the apoptotic cells labeled by green fluorescence. As shown in Figures 4(c) and 4(d), the number of TUNEL-positive cells significantly increased in the H_2_O_2_ group, which is consistent with the flow cytometry results. Preincubation with 1,25(OH)_2_D_3_ markedly attenuated the rate of apoptosis (*P* < 0.05), whereas LY294002 eliminated the protective effect of 1,25(OH)_2_D_3_ against H_2_O_2_-induced apoptosis (*P* < 0.05).

**Figure 5:**
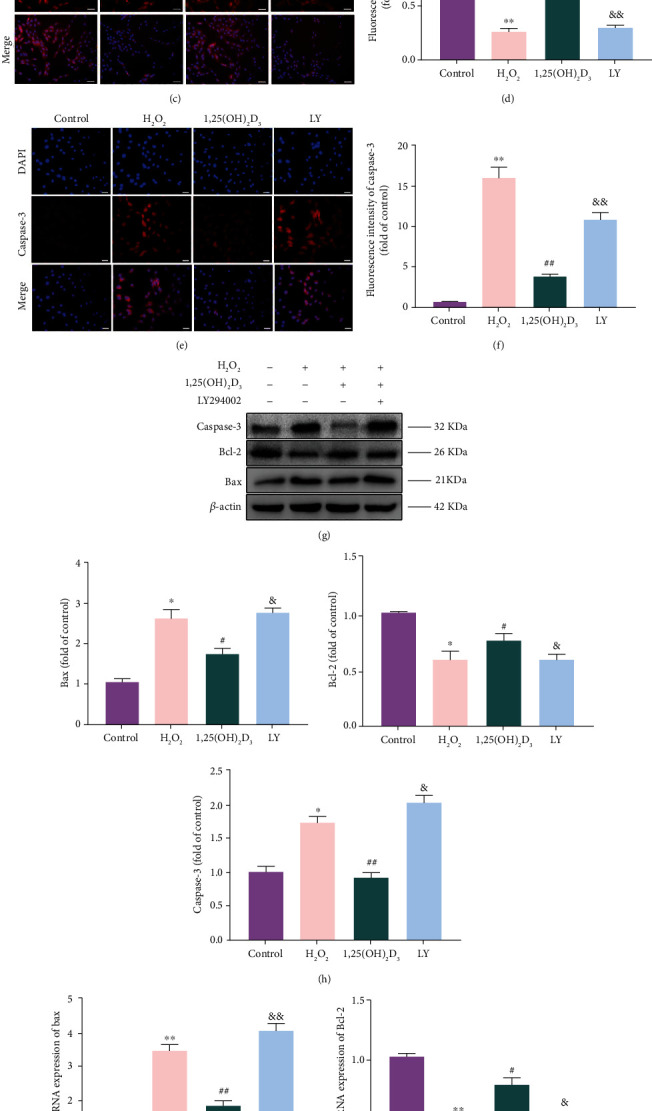
1,25(OH)_2_D_3_ upregulates the expression of Bcl-2 and downregulates the expression of Bax and Caspase-3. (a, b) Immunofluorescence staining and quantitative analysis of Bax (scale bar = 50 *μ*m). (c, d) Immunofluorescence staining and quantitative analysis of Bcl-2 (scale bar = 50 *μ*m). (e, f) Immunofluorescence staining and quantitative analysis of Caspase-3 (scale bar = 25 *μ*m). Immunofluorescence staining was observed by a fluorescence microscope. (g, h) The protein expressions and quantitative analysis of Bcl-2, Bax, and Caspase-3 in the different groups. (i) Quantitative analysis of the mRNA expression levels of Bcl-2, Bax, and Caspase-3. All data are the mean ± SD. ^∗^*P* < 0.05 and ^∗∗^*P* < 0.01 compared with the control group; ^#^*P* < 0.05 and ^##^*P* < 0.01 compared with the H_2_O_2_ group; ^&^*P* < 0.05 and ^&&^*P* < 0.01 compared with the 1,25(OH)_2_D_3_ group. LY: LY294002 group.

Immunofluorescence, western blotting, and qRT–PCR were adopted to assess the intracellular fluorescence, proteins, and mRNA expression, respectively, of the antiapoptotic molecule Bcl-2 and the proapoptotic molecules Bax and Caspase-3. The results showed that compared with the control group, Bax and Caspase-3 were significantly upregulated, but Bcl-2 was downregulated in NPMSCs of the H_2_O_2_ group (*P* < 0.05), whereas the fluorescence intensity, proteins, and mRNA expression levels of Bax and Caspase-3 were downregulated and that of Bcl-2 was upregulated in NPMSCs of the 1,25(OH)_2_D_3_ group compared with those in the H_2_O_2_ group (*P* < 0.05). However, the protective effect of 1,25(OH)_2_D_3_ on H_2_O_2_-induced apoptosis-related protein and mRNA expression was eliminated in the presence of LY294002 (*P* < 0.05, Figure 5).

### 3.6. Protective Effects of 1,25(OH)_2_D_3_ on Mitochondrial Homeostasis

**Figure 6:**
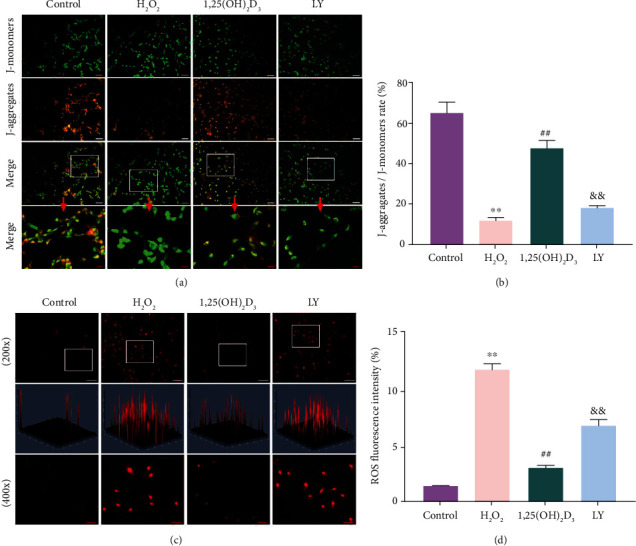
1,25(OH)_2_D_3_ protects mitochondrial membrane potential (MMP) and reduces reactive oxygen species (ROS) in nucleus pulposus-derived mesenchymal stem cells (NPMSCs). (a) Fluorescence results of MMP in the different groups. Mitochondrial aggregate JC-1 emits red fluorescence, and monomer JC-1 emits green fluorescence (white scale bar = 100 *μ*m; red scale bar = 25 *μ*m). (b) Quantitative analysis of MMP fluorescence intensity. (c) Fluorescence results of ROS in the different groups. A stronger red fluorescence indicates a higher ROS level. (d) Quantitative analysis of ROS fluorescence results. All data are the mean ± SD. ^∗∗^*P* < 0.01 compared with the control group; ^##^*P* < 0.01 compared with the H_2_O_2_ group; ^&&^*P* < 0.01 compared with the 1,25(OH)_2_D_3_ group. LY: LY294002 group.

The destruction of MMP is a landmark event in the early stage of mitochondrial homeostasis disorder and cell apoptosis. The polarized MMP was stained with orange–red fluorescence in the control group, but the orange–red fluorescence intensity was attenuated and the green fluorescence intensity was strengthened in the H_2_O_2_ group. Compared with the H_2_O_2_ group, the MMP of the 1,25(OH)_2_D_3_ group was still in the orange–red polarization state. However, the protective effect of 1,25(OH)_2_D_3_ on the cells was inhibited in the LY294002 group (Figures 6(a) and 6(b)). In addition, the excessive production of ROS can damage mitochondrial homeostasis. As shown in Figures 6(c) and 6(d), compared with the control group, the level of intracellular ROS in the H_2_O_2_ group was significantly increased (*P* < 0.05). However, the excessive production of ROS was significantly suppressed by preincubation with 1,25(OH)_2_D_3_ (*P* < 0.05), and the weakening effect of 1,25(OH)_2_D_3_ on ROS levels was decreased by preincubation with LY294002 (*P* < 0.05). These results represent that 1,25(OH)_2_D_3_ effectively alleviates H_2_O_2_-induced oxidative stress by diminishing the level of ROS in NPMSCs, and this effect was weakened by LY294002 implicating PI3K is involved in this process.

### 3.7. Effects of 1,25(OH)_2_D_3_ on the PI3K/Akt Pathway

**Figure 7:**
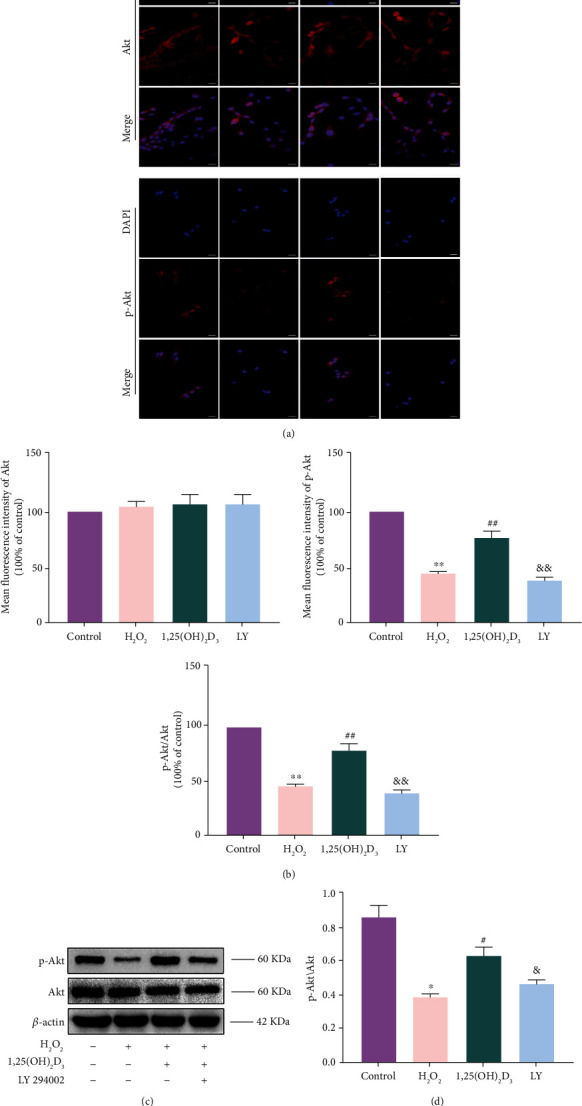
1,25(OH)_2_D_3_ alleviates the inhibition of H_2_O_2_ on the PI3K/Akt pathway. (a) Immunofluorescence staining of Akt and p-Akt in nucleus pulposus-derived mesenchymal stem cells (NPMSCs) is observed by a laser scanning confocal microscope (scale bar = 25 *μ*m). (b) Quantitative analysis of the fluorescence expressions of Akt and p-Akt. (c, d) The protein expression and quantitative analysis of Akt and p-Akt in the different groups. All data are the mean ± SD. ^∗^*P* < 0.05 and ^∗∗^*P* < 0.01 compared with the control group; ^#^*P* < 0.05 and ^##^*P* < 0.01 compared with the H_2_O_2_ group; ^&^*P* < 0.05 and ^&&^*P* < 0.01 compared with the 1,25(OH)_2_D_3_ group. LY: LY294002 group.

To further explore the mechanism of 1,25(OH)_2_D_3_ on NPMSC apoptosis, immunofluorescence and western blotting were used to evaluate the expression levels of Akt and p-Akt in the cytoplasm. The fluorescence intensity and protein expression of Akt among the four groups were not significantly different (*P* > 0.05). However, the fluorescence intensity and protein expression levels of p-Akt/Akt in the H_2_O_2_ group were significantly decreased compared with those in the control group (*P* < 0.05). Interestingly, this effect was significantly reversed by pretreatment with 1,25(OH)_2_D_3_ (*P* < 0.05), but pretreatment with LY294002 decreased the protective effect of 1,25(OH)_2_D_3_ by downregulating p-Akt in NPMSCs (*P* < 0.05, Figure 7). Our results demonstrate that the protective action of 1,25(OH)_2_D_3_ is related to activation of the PI3K/Akt pathway.

### 3.8. Radiographic and MRI Evaluation

**Figure 8:**
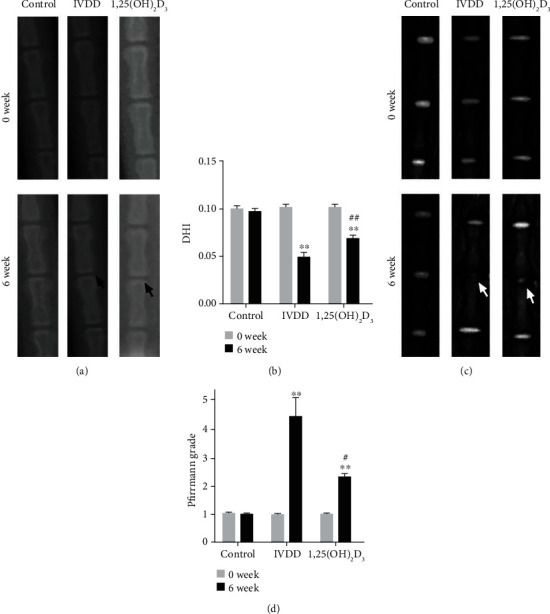
X-ray and magnetic resonance imaging (MRI) evaluation in intervertebral disc degeneration (IVDD) model at 0-week and 6-week after puncturing. (a, b) The X-ray scans and quantitative analysis of disc height index (DHI) in the different groups (control group, IVDD group, and 1,25(OH)_2_D_3_ group). (c, d) The MRI images and quantitative analysis of Pfirrmann grades in the different groups. All data are the mean ± SD. ^∗∗^*P* < 0.01 compared with the control group; ^#^*P* < 0.05 and ^##^*P* < 0.01 compared with the IVDD group.

X-ray was performed to evaluate DHI, and no significant difference in DHI was found between the three groups before puncture. However, compared with the control group (0.0983 ± 0.037), the DHI of the IVDD group (0.050 ± 0.004) was significantly lost at 6 weeks postpuncture (*P* < 0.01). Furthermore, the DHI of the 1,25(OH)_2_D_3_ group (0.070 ± 0.003) was higher than that of the IVDD group (*P* < 0.01) (Figures 8(a) and 8(b)). Moreover, the degenerative grade of IVD as measured by MRI according to Pfirrmann classification showed that the Pfirrmann grade of the three groups before puncture was no statistically significant difference. However, at six-week postpuncture, the grade of the IVDD group was significantly more serious than that of the control group (*P* < 0.05), and the grade of the 1,25(OH)_2_D_3_ group was slighter than that of the IVDD group (*P* < 0.05, Figures 8(c) and 8(d)). The results showed that 1,25(OH)_2_D_3_ intervention could delay the progression of IVDD *in vivo*.

### 3.9. Histological Analysis

**Figure 9:**
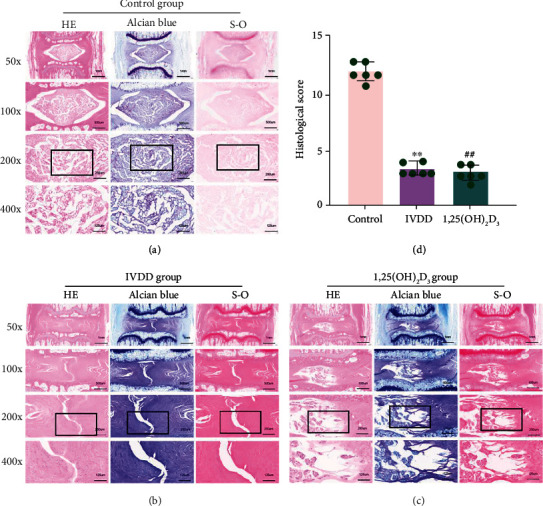
Hematoxylin-eosin (HE), toluidine blue, and safranin-O/fast green (S-O) staining. (a-c) The staining at 6 weeks after a puncture in the control group, intervertebral disc degeneration (IVDD) group, and 1,25(OH)_2_D_3_ group. (d) Quantitative analysis of histological score in the different groups. All data are expressed as the mean ± SD. ^∗∗^*P* < 0.01 compared with the control group; ^##^*P* < 0.01 compared with the IVDD group.

HE, toluidine blue, and S-O staining of the control group showed well-structured inner gel-like NP and outer concentric ring-like AF tissues. In contrast, the NP tissue almost disappeared, and the well-structured IVD tissue was damaged in the IVDD group; the histological score was also significantly lower than that of the control group (*P* < 0.01). Interestingly, there was still some NP tissue in the 1,25(OH)_2_D_3_ group, and the histological score was higher than that of the IVDD group (*P* < 0.01, Figure 9).

## 4. Discussion

LBP has become the most common cause of disability in adults worldwide in the past 30 years, and IVDD is an important cause of LBP in middle-aged and elderly patients. As a result, the treatment cost places a substantial burden on the family and society [2, 3]. Much effort has been expended in the development of medicines and surgical treatments for IVDD, but they mainly relieve symptoms and cannot effectively alleviate or reverse the process of IVDD [34, 35]. In recent years, MSC-based biological treatment has gradually exhibited advantages [6–9, 25]. However, previous studies found that the local environment of the degenerated IVD, which is characterized by nutrient deficiency, hypertonicity, low pH, hypoxia, and high mechanical loading, can decrease the number and functionality of the transplanted MSCs [11, 36, 37]. Additionally, MSC transplantation has some potential side effects such as osteophyte formation due to MSC migration [38].

Interestingly, previous studies including ours have confirmed that endogenous NPMSCs exist in normal and degenerated IVDs [23–25]. The NPMSCs isolated from NP tissues in the present study also showed characteristics that fulfilled the criteria outlined by the ISCT for MSC [27]. Furthermore, NPMSCs exhibited more potent biological activity than other tissue-derived MSCs in the hypoxic environment of IVD [39]. NPMSCs may enhance the repair and regeneration ability of degenerative IVDs by differentiating into NP cells and/or inhibiting NP cell apoptosis [18, 24, 25], but the number of NPMSCs gradually decreases with the degeneration of the IVD, which may lead to the failure of endogenous repair [40]. Therefore, it is particularly important to maintain the number of viable and functional NPMSCs for the process of endogenous repair of IVDD.

The active metabolite of vitamin D, 1,25(OH)_2_D_3_, is a fat-soluble vitamin and a steroid compound, and the gene encoding its intracellular specific receptor (VDR) is the first reported gene that may be related to the risk of IVDD [41]. Also, 1,25(OH)_2_D_3_ has shown broad protective effects such as anti-inflammatory, antioxidant, antisenescence, and antiapoptosis [42–44]. Thus, the present study evaluated the effect of 1,25(OH)_2_D_3_ on H_2_O_2_-induced oxidative damage in NPMSCs for the first time and discussed its possible mechanism.

Although 1,25(OH)_2_D_3_ has shown a broad protective effect, there is still a controversy about the effective concentration. A previous study found that 1,25(OH)_2_D_3_ has a positive effect on cartilage cell proliferation at a concentration of 10^−12^ M but a dose-dependent inhibitory effect at 10^−10^ M and above [45]. Colombini et al. [17] and Gruber et al. [46] found that 10^−8^ M 1,25(OH)_2_D_3_ significantly inhibits the proliferation of AF cells and NP cells. More fascinating was the result reported by Klotz et al., where 10^−7^ M 1,25(OH)_2_D_3_ treatment for 72 h not only significantly inhibited BMSC proliferation but also inhibited cell apoptosis. In addition, it also delayed the development of replicative senescence in long-term culture. Moreover, BMSCs maintained clonogenic capacity, stem cell surface marker characteristics, and multipotent differentiation capacity after long-term 1,25(OH)_2_D_3_ treatment [47]. Our study found that 1,25(OH)_2_D_3_ showed no cytotoxic effect on cell viability at concentrations of 10^−8^ M to 10^−12^ M, and 10^−10^ M showed the most obvious protective effect on cell proliferation. Therefore, a concentration of 10^−10^ M was used to further explore the effect of 1,25(OH)_2_D_3_ on the biological process of NPMSCs.

Cell senescence exhibits the characteristics of decreased cell viability and the loss of proliferation capacity. Our present results found that pretreatment with 1,25(OH)_2_D_3_ can significantly decrease the ratio of oxidative stress-induced cell senescence and apoptosis, and the inhibited proliferation was also significantly mitigated, although it did not improve to the level of normal NPMSCs. Huang et al. and Zhang et al. also found that 1,25(OH)_2_D_3_ inhibits NP cell apoptosis via the regulation of the nuclear factor-*κ*B (NF-*κ*B) signaling pathway [42, 48]. Tong et al. found that 1,25(OH)_2_D_3_ can activate intracellular VDR in AF cells, and activation of VDR ameliorates oxidative stress-induced apoptosis by preserving mitochondrial function [43]. Bcl-2 (antiapoptotic protein) and Bax (proapoptotic protein) are the two proteins used to evaluate cell susceptibility to apoptosis. The activity of the caspase pathway would be promoted by Bax but inhibited by Bcl-2 and inhibit cell apoptosis [49]. The present study also found that 1,25(OH)_2_D_3_ significantly inhibited the upregulated expression levels of p53, p21, Caspase-3, and Bax induced by H_2_O_2_ and restored the downregulated expression level of Bcl-2. Therefore, these results suggest that 1,25(OH)_2_D_3_ could protect NPMSCs from H_2_O_2_-induced damage.

The accumulation of ROS leads to the destruction of mitochondrial homeostasis by intensifying the opening of the mitochondrial permeability transition pore and the release of cytochrome *c*. Subsequently, caspase-dependent cell apoptosis was activated [50]. The formation and development of IVDD are tightly associated with the presence of ROS, which is regarded as influential intermediates in the pathogenesis of IVDD [14, 24, 51]. Similar to a previous study [18], our present study also found that the proliferation ability and MMP of NPMSCs were inhibited under oxidative stress induced by H_2_O_2_, and ROS production and cell apoptosis rates were increased. These results further confirm that oxidative stress is harmful to maintaining the healthy physiology of NPMSCs. A previous study found that 1,25(OH)_2_D_3_ treatment significantly reduces the level of oxidative stress in degenerated IVD tissue [42]. Additionally, Tong et al. also found that 1,25(OH)_2_D_3_ treatment for 12 h can significantly reduce the increased ROS level caused by H_2_O_2_ and increase the ratio of JC-1 polymer to monomers in rat AF cells [43]. Notably, our results also found that the increased ROS level and upregulated J-aggregates of MMP induced by H_2_O_2_ in NPMSCs were significantly inhibited by 1,25(OH)_2_D_3_. Therefore, these results demonstrate that 1,25(OH)_2_D_3_ may exert a positive effect on antioxidative stress and may regulate the function of mitochondria to protect NPMSCs.

**Figure 10:**
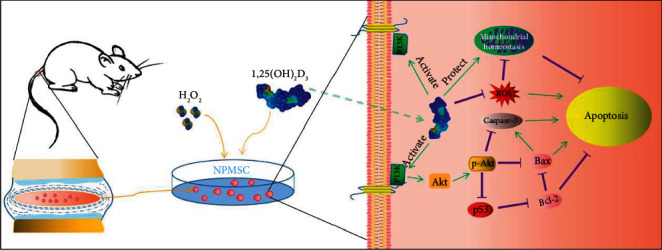
Schematic of the protective effects of 1,25(OH)_2_D_3_. 1,25(OH)_2_D_3_ activates PI3K/Akt pathway to facilitate antioxidation, enhance mitochondrial homeostasis, and alleviate nucleus pulposus-derived mesenchymal stem cell (NPMSC) apoptosis *in vitro*.

The PI3K/Akt pathway plays a key role in modulating biological processes. The activated PI3K/Akt pathway participates in the processes of cell proliferation, apoptosis, and differentiation through interactions with downstream proteins under physiological and pathological conditions [18–22, 52–54]. Previous studies have found that the PI3K/Akt pathway participates in the proliferation and apoptotic processes of NPMSCs [18–20, 24], and 1,25(OH)_2_D_3_ can modulate biological processes through PI3K/Akt pathway [21, 53]. The present results also found that 1,25(OH)_2_D_3_ could partially alleviate the inhibition of the PI3K/Akt pathway induced by H_2_O_2_, thereby reducing ROS production and improving the MMP. However, the positive effect of 1,25(OH)_2_D_3_ on NPMSCs was weakened when the PI3K/Akt pathway was inhibited by LY294002. Therefore, as shown in Figure 10, our results indicate that 1,25(OH)_2_D_3_ may reduce H_2_O_2_-induced apoptosis of NPMSCs by activating the PI3K/Akt pathway.

To further evaluate the protective effect of 1,25(OH)_2_D_3_, 1,25(OH)_2_D_3_ was used in the IVDD animal model. The results found that 1,25(OH)_2_D_3_ can partially reduce the loss of intervertebral space and delay the degree of IVDD. HE, toluidine blue, and S-O histological staining also showed that 1,25(OH)_2_D_3_ can defer the loss of NP tissue, which further confirmed that 1,25(OH)_2_D_3_ has a positive effect on postponing IVDD in the animal model.

This study also has several limitations. First, the NPMSCs were cultured under normal oxygen conditions, which is not the actual physiological condition of IVDD. Second, 1,25(OH)_2_D_3_ was found to participate in various metabolic processes and regulate cell biological processes via multiple signaling pathways, such as nuclear factor (erythroid-derived 2)-like 2 (Nrf2), NF-*κ*B [42, 44, 48]. Therefore, further study is needed to explore the other mechanisms underlying the protective effect of 1,25(OH)_2_D_3_ on NPMSCs.

## 5. Conclusions

In conclusion, this is the first research on the protective action of 1,25(OH)_2_D_3_ against H_2_O_2_-induced oxidative damage in NPMSCs. The administration of 1,25(OH)_2_D_3_ not only activated the PI3K/Akt pathway to protect mitochondrial function and mitigate the apoptosis of NPMSC *in vitro* but also could retard IVDD *in vivo*. The results support 1,25(OH)_2_D_3_ as a promising treatment to repair and delay IVDD.

## Figures and Tables

**Table 1:** Sequences of primers used for real-time PCR.

Gene	Primer/probe sequence
*β*-Actin	Forward 5′-TTGTAACCAACTGGGACGATATGG-3′ Reverse 5′-GATCTTGATCTTCATGGTGCTAGG-3′
Caspase-3	Forward 5′-GGCCTGCTTTTTACCTCAGA-3′ Reverse 5′-CGTTTCCGCACAGGCTGCTT-3′
Bcl-2	Forward 5′-GCGTCAACAGGGAGATGTCA-3′ Reverse 5′-GGTATGCACCCAGAGTGATG-3′
Bax	Forward 5′-GGTTTCATCCAGGATCGAGACG-3′ Reverse 5′-ACAAAGATGGTCAGGGCTTGCC-3′

## Data Availability

All data during the study are available from the corresponding author by request.
